# Presence of state transitions in the cryptophyte alga *Guillardia theta*

**DOI:** 10.1093/jxb/erv362

**Published:** 2015-08-06

**Authors:** Otilia Cheregi, Eva Kotabová, Ondřej Prášil, Wolfgang P. Schröder, Radek Kaňa, Christiane Funk

**Affiliations:** ^1^Department of Chemistry, Umeå University, SE-90187 Umeå, Sweden; ^2^Institute of Microbiology, Centre Algatech, Laboratory of Photosynthesis, Opatovický Mlýn, Třeboň 379 81, Czech Republic

**Keywords:** Blue/low light adaptation, chlorophyll a/c antenna, cryptophytes, growth stage, non-photochemical quenching, state transitions.

## Abstract

Plants and algae have developed various regulatory mechanisms for optimal delivery of excitation energy to the photosystems even during fluctuating light conditions; these include state transitions as well as non-photochemical quenching. The former process maintains the balance by redistributing antennae excitation between the photosystems, meanwhile the latter by dissipating excessive excitation inside the antennae. In the present study, these mechanisms have been analysed in the cryptophyte alga *Guillardia theta*. Photoprotective non-photochemical quenching was observed in cultures only after they had entered the stationary growth phase. These cells displayed a diminished overall photosynthetic efficiency, measured as CO_2_ assimilation rate and electron transport rate. However, in the logarithmic growth phase *G. theta* cells redistributed excitation energy via a mechanism similar to state transitions. These state transitions were triggered by blue light absorbed by the membrane integrated chlorophyll a/c antennae, and green light absorbed by the lumenal biliproteins was ineffective. It is proposed that state transitions in *G. theta* are induced by small re-arrangements of the intrinsic antennae proteins, resulting in their coupling/uncoupling to the photosystems in state 1 or state 2, respectively. *G. theta* therefore represents a chromalveolate algae able to perform state transitions.

## Highlight

The cryptophyte alga *Guillardia theta* performs state transitions and photoprotection. The mechanism is dependent on its chlorophyll *a/c* antenna and the growth state of the culture.

## Introduction

Harvesting of sun light is the first step in the photosynthetic process. In the photosynthetic light reaction, antenna complexes (reviewed by Jansson, 1999; Dall’Osto *et al.*2015) rapidly transfer absorbed light energy to a reaction center, which is located in a special membrane system called thylakoid membrane. While the reaction centers of Photosystem II (PSII) and Photosystem I (PSI) remained highly conserved during evolution, various antenna systems have evolved in photosynthetic organisms. *Prokaryotic cyanobacteria and eukaryotic red algae contain phycobilisomes with covalently bound phycobilin-pigments as their major antennae* (Bailey *et al.*, 2008). These structures are extrinsically associated to the outside of the thylakoid membrane. In higher plants and green algae, the most abundant antenna is the chlorophyll *a*/*b*-binding light harvesting complex (referred to as LHC), which is inserted into the thylakoid membrane (Neilson and Durnford, 2009). Cryptophytes acquired their chloroplasts by secondary endosymbiogenesis (Gibbs, 1981) and, similary to red algae, the progenitor of their chloroplasts, use two different light-harvesting systems: phycobiliproteins and chlorophyll *a/c*-binding proteins. The Chl *a/c*- binding proteins are homologous to the Chl *a/b* of plants (Green and Durnford, 1996; Durnford *et al.*, 1999). Even though the phycobiliproteins originated from the red algal ancestor, in cryptophytes they are not organized into phycobilisomes bound to the stromal face of the thylakoids, but are located in the thylakoid lumen (Spear-Bernstein and Miller, 1989). Variations in the lumen-exposed domains of the Chl *a/c* proteins may have implications for interaction between the intrinsic light-harvesting antennas and the lumen-located, soluble phycobiliprotein (Broughton *et al.*, 2006).

Light harvesting provides a major challenge in optimizing photosynthesis; while light provides the energy necessary for carbon fixation, at the same time it damages the cells. When more light is absorbed than can be used for photochemistry, the excess energy can potentially lead to the production of highly reactive oxygen species that cause photo-oxidative damage and inhibit photosynthesis (Li *et al.*, 2009). Photosynthetic organisms have adapted by evolving a variety of direct and indirect protection mechanisms. Long-term acclimations to the prevalent light intensity involve regulation of the antenna size (Kopečná *et al.*, 2012; Kouřil *et al.*, 2013). However, besides adjusting light absorption, photosynthetic organisms can dissipate excess light energy in a process called non-photochemical quenching (NPQ) (de Bianchi *et al.*, 2010). The molecular mechanisms of NPQ vary among cyanobacteria, algae, and higher plants depending on their different light-harvesting antennae (reviewed in Niyogi and Truong, 2013; Kirilovsky *et al.*, 2014; Goss and Lepetit, 2015), still, the final outcome is the same: excess excitation energy is dissipated as heat (Kaňa and Vass, 2008). The main component of NPQ (for recent review see e.g. Ruban *et al.*, 2012) is the energy-dependent quenching (qE), also called feedback de-excitation (Li *et al.*, 2000). Another rapid physiological mechanism for adaptation to light quality and intensity are state transitions. They allow balancing of the absorption capacity of the two photosystems by reversible relocation of the light-harvesting complexes between PSII to PSI (Dietzel and Pfannschmidt, 2008). Generally, light leading to excess excitation of PSII induces a transition to state 2, in which excitations are diverted towards PSI. On the contrary, when PSI is over-excited the state 1 is induced, transferring light energy to PSII. State transitions are well documented in plants and green algae (reviewed by Minagawa, 2011) as well as in cyanobacteria (Campbell *et al.*, 1998; Kirilovsky *et al.*, 2014), while state transitions in algae from the red clade have been analysed less extensively (Owens, 1986; Snyder and Biggins, 1987; Gibbs and Biggins, 1989; Delphin *et al.*, 1996). The first proposal of state transition in red algae was made by the Norio Murata (Murata, 1969), however its mechanism is still not clear (Delphin *et al.*, 1995). A state-transition-’like’ behavior in cryptophytes has been observed in *Cryptomonas ovata*; energy delivering to the photosystems was controlled by small conformational changes/uncoupling of the pigment-proteins and considered to be the major photoprotective mechanism in cryptophytes (Snyder and Biggins, 1987). Recently, other photoprotective mechanisms have been studied in various cryptophytes (Funk *et al.*, 2011; Kaňa *et al.*, 2012b). Cryptophytes are adapted to very low light and are able to take advantage of quantum coherence to improve the efficiency of energy capturing (Collini *et al.*, 2010; Harrop *et al.*, 2014). In *Rhodomonas salina* a new type of NPQ has been described (Kaňa *et al.*, 2012b) and recently it was shown that *Guillardia theta* is able to perform non-photochemical quenching (Funk *et al.*, 2011). Here we performed in-depth analysis of the photoprotection mechanisms and photochemistry of *G. theta*. It is shown that the mechanisms of photoprotection and photosynthetic efficiency change significantly with culture age. In the logarithmic growth phase, light harvesting is accompanied by a state transitions-like mechanism and the absorbed light is efficiently used for carbon fixation. Blue light absorbed by the chlorophyll *a/c* antennae induces these state transitions, while green light, exciting the lumen-localized phycoerythrins, has no stimulating effect. In the stationary growth phase no state transitions take place, instead non-photochemical quenching is induced, leading to a drastic decrease of photosynthetic activity. The results show that the cryptophyte algae *G. theta* displays a growth phase-dependent photoprotection mechanism and highlight the presence of state transitions in cryptophytes.

## Materials and methods

### Culturing and cell counting

*G. theta* cells (CCMP2712) were obtained from the Provasoli-Guillard National Center for Culture of Marine Phytoplankton. Cultures were grown in Fernbach culture flasks in h/2 media (Guillard, 1975) shaken (120rpm) at 18 °C under white light (30 µmol photons m^−2^ s^−1^) with light-dark cycle (12 h:12h). *Rhodomonas salina* (strain CCAP 978/27) was grown in the same media and at the same conditions as *G. theta*.

Cultures of one litre were started with the same number of cells (~10^5^). Every second day cell number and size were determined using a calibrated Coulter Counter (Beckman Multisizer III) equipped with a 70 μm aperture. Samples were measured in triplicate.

### Pigment determination

Chlorophyll *a* and *c* concentrations in the cells were determined by absorption using an UV/VIS spectrophotometer (Unicam UV 550, Thermo Spectronic, UK) and calculated according to the equations of Jeffrey and Humphrey (1975). Triplicates of 10–15ml of the algal suspension were filtered onto Whatman GF/F filters; the pigments were extracted by 90% acetone for 24h at 4 °C in darkness.

### Absorption and fluorescence spectra

Absorption spectra were recorded by a spectrophotometer (Unicam UV 550, Thermo Spectronic, UK) equipped with an integrating sphere. Cells were collected on nitrocellulose membrane filters (Pragochema, Czech Republic) and the filters were then positioned in the integrating sphere. Absorbance was measured between 400 and 800nm, with a bandwidth of 4nm.

Fluorescence emission spectra (77 K) were measured using an Aminco-Bowman Series 2 spectro-fluorometer (Thermo Fisher Scientific, USA). Cells (3ml) adapted at room temperature either to white low light (2.5 µmol photons m^−2^ s^−1^) for 10min or to darkness for 20min, were filtered onto Whatman GF/C filters, which then were fitted in a sample holder and immersed in an optical Dewar flask filled with liquid nitrogen. Fluorescence was excited at 436 or 530nm and measured from 550 to 800nm with a bandwidth of 2nm.

### Measurements of non-photochemical quenching

Chl *a* fluorescence quenching was performed using a double-modulation FL100 fluorometer (Photon System Instruments, Brno, Czech Republic). Non-photochemical quenching (NPQ) was measured with cells in high-fluorescence state 1, induced by low intensity white light (2.5 µmol photons m^−2^ s^−1^, for 10min). The maximal fluorescence of low-light adapted cells (*F*_m_) was measured by a blue (464nm) multiple-turnover saturating flash (duration 350ms, intensity 4000 µmol photons m^−2^ s^−1^). The presence of high-fluorescence state 1 was tested using a short period of low-intensity blue light (464nm, 7 µmol photons m^−2^ s^−1^) when the flash was repeated before and after the light period. The extent and kinetics of non-photochemical quenching were studied in these cells during high-intensity actinic blue light treatment (464nm at 750 µmol photons m^−2^ s^−1^, for 150 s); a series of blue multiple-turnover saturating flashes was applied to obtain the *F*_m_′ value. Recovery kinetics of NPQ was measured during exposure to low-intensity blue light (464nm, 7 µmol photons m^−2^ s^−1^) with series of multiple-turnover saturating flashes to obtain *F*_m_′′ values during NPQ recovery. The non-photochemical quenching (NPQ) was calculated based on the Stern–Volmer formula NPQ =(Fm−Fm′)/Fm′ Extent of NPQ during growth phases was determined from the NPQ value obtained after 150 s of blue actinic light.

### Measurements of effective cross-section of PSII

Fast repetition rate fluorescence (FRRF) was used to estimate effective PSII antennae cross-section (σPSII), PSII connectivity (p) and electron transport rate (ETR). The method uses single turnover flashes, which are fitted according the model of Kolber and co-workers (Kolber *et al.*, 1998) to calculate the σPSII and *p*-values. All experiments were carried out at 18 °C with a custom-designed FL3500 fluorometer (Photon Systems Instruments, Brno, Czech Republic). Before the measurements the cells were kept under low intensity white light illumination (2.5 µmol photons m^−2^ s^−1^) for 10min to induce the high-fluorescent state 1. Single turnover flashes were induced by application of a series of 100 blue (463nm) flashlets of 1 μs duration. Single turnover flashes were applied during sequential exposure to background actinic light (590nm, 11 steps with increasing intensities, 0–1415 µmol photons m^−2^ s^−1^), fluorescence was detected at 680–700nm. ETR was calculated for every actinic irradiation step as follows:

$$ETR=\sigma PSII\;\times \;n_{\text{PSII}}\;\times \;(F_{\text{q}}{´}_{\text{s}}/F_{\text{v}}{´}_{\text{s}})/(F_{\text{vs}}/F_{\text{ms}})\;\times \;I$$

where *F*_ms_ (*F*_m_′_s_) is the maximal fluorescence at state 1 in the dark (*F*_ms_) or in the light (*F*_m_′_s_), *F*_vs_ the variable fluorescence in the dark, *F*_q_′_s_ equals *F*_m_′_s_-*F*′, where *F*′ represents steady state value of fluorescence at a given irradiance, I is the light intensity and n_PSII_ gives the ratio of functional PSII to total chlorophyll *a.* The value $1/n_{\text{PSII}}=500$ [mol chl*a*/mol RCII] was used, which had been previously estimated for cryptophytes [see Suggett *et al.*, (2010) for details].

State transitions were studied using a Double-modulation FL100 fluorimeter (Photon System Instruments, Brno, Czech Republic). Before the measurement, cell suspensions (2ml) were placed in a stirred cuvette and dark adapted for 20min to ensure transition to low fluorescence state 2. The minimal value of fluorescence (*F*_0_) was then measured immediately after dark adaptation in low-intensity blue measuring light. Dark adapted cells were illuminated with low-intensity blue light (7 µmol photons m^−2^ s^−1^) to induce the transition from state 2 to state 1. Afterwards, the reverse process (transition from state 1 to state 2) was induced by keeping cells in dark, the maximal fluorescence *F*_m_′ was measured by the same multiple turnover flashes (464nm, duration 350ms, intensity 4200 µmol photons m^−2^ s^−1^) applied either every 20 s or on the end of the dark period. The ability to perform state transition during growth was measured every 2 d from the ratio $F_{\text{v}}(\text{LL})/F_{0}(\text{LL})-F_{\text{v}}(\text{D})/F_{0}(\text{D})$. Parameters *F*_v_(LL) and *F*_v_(D) represent variable fluorescence values measured with cells adapted to 2 µmol photons m^−2^ s^−1^ of white low-light for 10min (LL) and cells taken from dark (D), respectively. To remove the effect of increasing culture density during the growth, the *F*_v_ values were normalized by dividing by respective minimal fluorescence levels, *F*_0_(LL) or *F*_0_(D).

### Kinetic fluorescence spectroscopy

Changes in fluorescence emissions during state transitions were measured using the spectrally resolved fluorescence induction (SRFI) method (see e.g. Kaňa *et al.*, 2012a; Kotabová *et al.*, 2014). In this method the diode-array spectrophotometer SM-9000 (Photon System Instrument, Brno, Czech Republic) was combined with a double-modulation FL100 fluorimeter (Photon System Instruments, Brno, Czech Republic) that was used for actinic light excitation and spectra synchronization. Before the measurement, cell suspensions were dark-adapted for 20min to ensure transition to the low fluorescence state 2. The spectral changes in maximal fluorescence were then detected before and after application of low-intensity blue light (464nm, 7 µmol photons m^−2^ s^−1^) for 80 s causing the transition from state 2 to state 1. Maximal fluorescence was induced by multiple-turnover flashes (464nm, duration 350ms, intensity 4200 µmol photons m^−2^ s^−1^).

### Photosynthesis

The photosynthetic carbon fixation was determined using the small volume ^14^C incubation method of (Lewis and Smith, 1983). Sample aliquots of 1ml were incubated for 40min at 18 °C with ^14^C-sodium bicarbonate (MP Biochemicals, USA) at a final concentration of 1 µCi m^l−1^ in the laboratory-built photosynthetron (Bruyant *et al.*, 2005). Samples for triplicate background counts (with 50 µλ of formaldehyde) and total counts (with 50 µλ of ethanolamine) were prepared. Samples with 50 % HCl (1ml) were left overnight to purge off unincorporated label before 10ml of Ecolume scintillation cocktail was added and disintegrations per minute were counted on a calibrated Tri-Carb 2810 TR Liquid Scintillation Analyser (Perkin Elmer, USA). Dissolved inorganic carbon concentrations were determined in a cell-free medium by the Gran titration technique described by (Butler, 1982). The carbon fixation rate was normalized to the Chl content.

## Results

### Characterization of growth phases in *G. theta* cultures

**Fig. 1.**
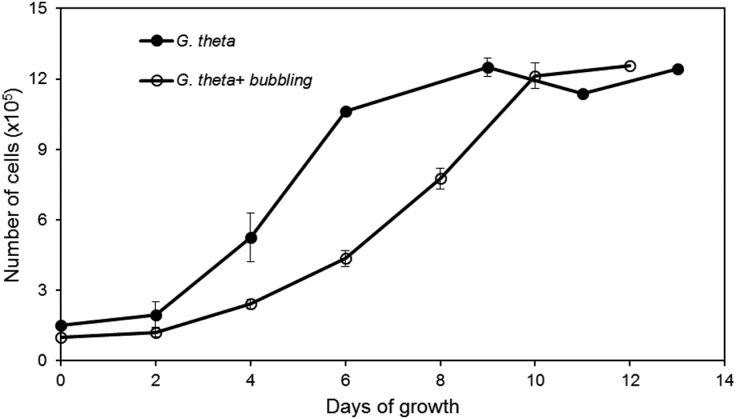
Growth curve of *G. theta* cells in shaken (closed circles) and air-bubbled (open circles) batch cultures. Cultures of one litre were started with the same number of cells (~10^5^); cell number was measured in triplicate.

Growth of *G. theta* cells in shaking batch culture (12h/12h day/night irradiance rhythm) was monitored over a period of 13 d; *G. theta* had a doubling time of 34h in its logarithmic phase (Days 2–6). However, already from Day 6 the growth rate slowed down and the culture entered the stationary phase (Fig. 1); characteristic time points are Day 2 (early logarithmic phase), Day 6 (turning point to stationary phase), Day 9 (early stationary phase), and Day 13 (late stationary phase). Cultures that were not shaken, but instead bubbled with air, had reduced growth rate and entered the stationary phase 2–3 d later, however, at roughly the same cell density compared with the shaken culture (Fig. 1). The growth curve of *G. theta* was compared with *Rhodomonas salina*, a related cryptophyte, often referred to as the model cryptomonad. Under the same growth conditions like *G. theta*, the growth of *R. salina* remained in logarithmic phase throughout the whole experimental period of 14 d (data not shown).

**Fig. 2.**
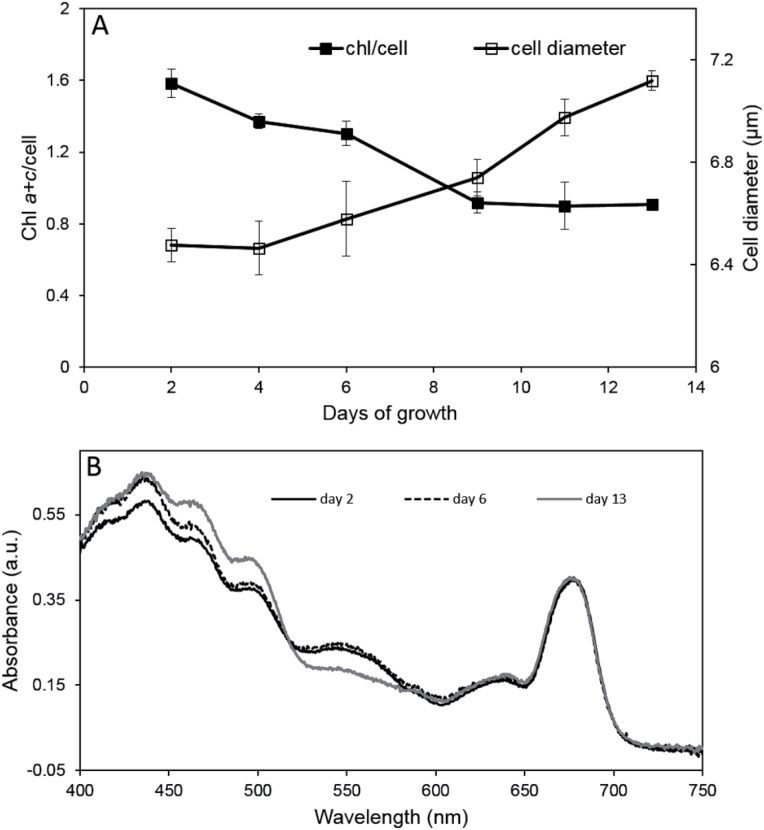
(A) Cell size (open squares) and amount of chlorophyll per cell (closed squares) of a *G. theta* batch culture grown under standard conditions (shaking) for 2 weeks. Triplicates were measured per time point. (B) Whole cells absorption spectra of a culture in logarithmic growth phase (Day 2, black line), at its turning point (Day 6, dotted black line), and in stationary phase (Day 13, grey line).

The diameter of individual *G. theta* cells increased gradually with culture age (Fig. 2A); when the cells entered the stationary phase at Day 6, with decreased doubling time the *G. theta* cells continued to grow in size. The total amount of chlorophyll per cell decreased during the logarithmic phase, but remained constant in the stationary phase (Fig. 2A). The amount of Chl *a* decreased by 43% from 1.34 pg/cell (Day 2 after culture started) to 0.750 pg/cell (in 13-d-old cultures), meanwhile Chl *c* only decreased from 0.234 pg/cell (Day 2) to 0.156 pg/cell (Day 13), resulting in a decreased Chl *a/*Chl *c* ratio by 13% during the experiment.

Changes in pigment composition and antennae organization during the growth phases were monitored by whole cell absorption spectra. Figure 2B shows whole cell absorption spectra of the culture in dependence of the growth phases: early logarithmic phase (Day 2), turning point to stationary phase (Day 6), and stationary phase (Day 13). The spectra were characterized by absorption maxima at 436nm (mainly Chl *a*), 465nm (Chl *c*), 495nm (alloxanthin), 545nm (phycoerythrin), and 680nm (Chl *a*). The carotenoid:Chl *a* ratio increased from 0.9 (logarithmic phase) to 1.2 (stationary phase), while the amount of phycoerythrin decreased in stationary phase.

**Fig. 3.**
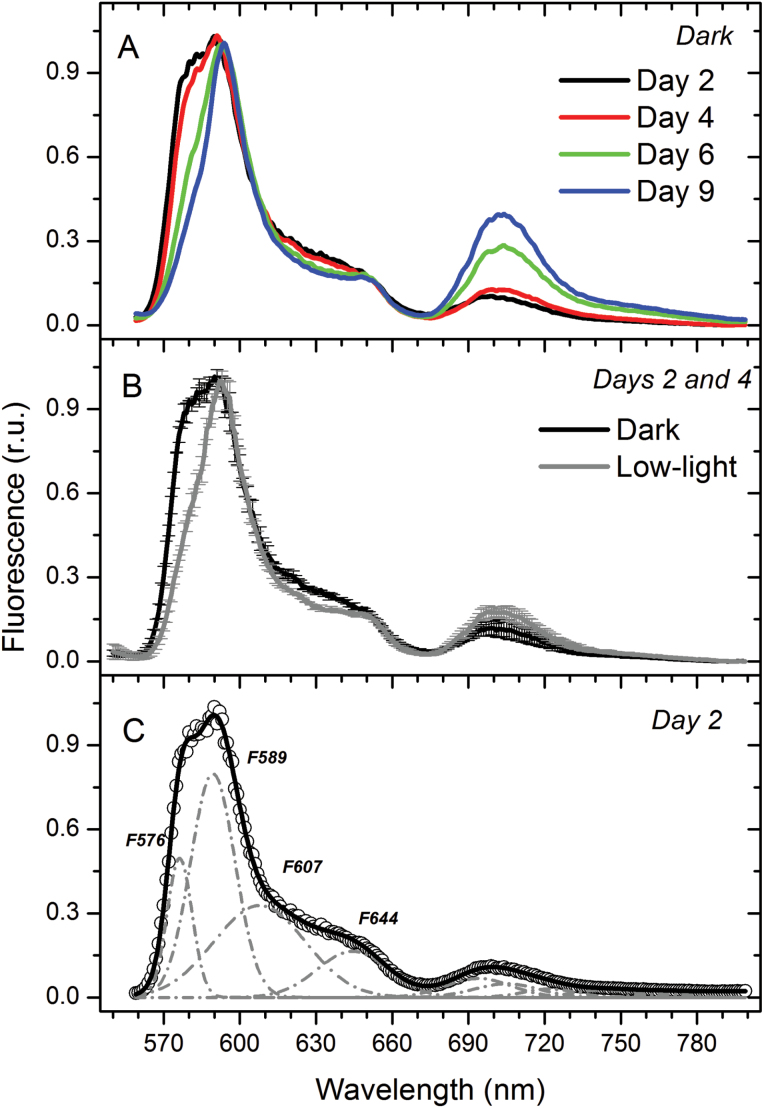
Low temperature (77 K) fluorescence emission of *G. theta* cells after excitation of phycoerythrin at 530nm. The spectra were normalized to the maximal phycoerythrin emission at 590nm. Displayed curves are an average of three measurements. (A) Spectra measured at different culture ages after 20min dark adaptation. (B) Average spectra obtained of the cultures in logarithmic phase (Days 2 and 4), either were dark-adapted for 20min (black curve) or exposed to low white light (2 µmol photons m^−2^ s^−1^) for 10min (grey curve). (C) Deconvoluted spectrum obtained of *G. theta* cells in logarithmic growth phase (Day 2), dark adapted for 20min. The four main fluorescence emission maxima (F576, F589, F607, and F644) are indicated.

Changes in the functional organization of phycoerythrin (PE) in *G. theta* cells were analysed by low temperature (77 K) fluorescence emission spectra (Fig. 3). De-convoluted spectra of dark adapted cells in the early logarithmic growth phase revealed two main PE emission bands, F576 and F589, and two additional minor bands, F607 and F644 (Fig. 3C). F576 almost disappeared in the stationary phase (Fig. 3A), while the fluorescence bands at 694nm (PSII) and 705nm (PSI) increased, indicating that F576 PE is energetically stronger coupled to PSII and PSI during the stationary growth phase. Interestingly, the largest F576 emission was observed in young cells that were dark adapted for 20min, fluorescence emission decreased in cells adapted to low white light (Fig. 3B).

### Light harvesting and photoprotective mechanisms of *G. theta* change age-dependently

**Fig. 4.**
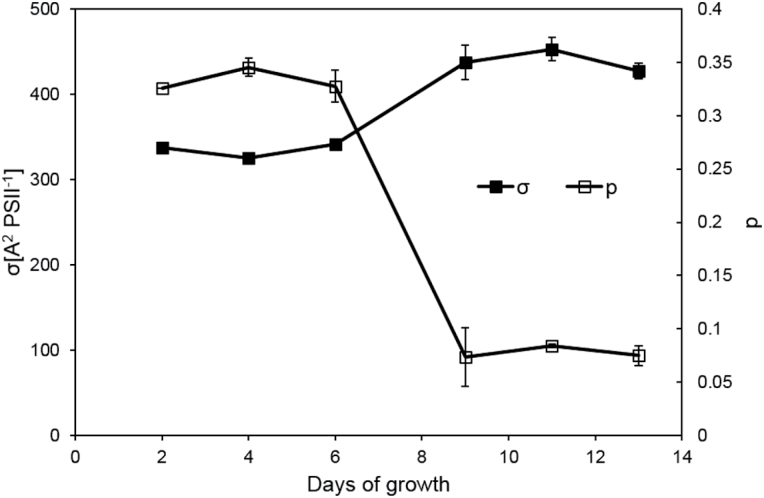
Connectivity of PSII reaction centres (p, open squares) and effective antenna cross-section of PSII (σ, closed squares) measured in *G. theta* cells in dependence of the culture age. All measurements were performed in triplicate and error bars represent standard deviation. Fluorescence during single-turnover was detected at 680–700nm.

The effective PSII antenna size (σ_PSII_) and connectivity of PSII reaction centers (*p*) was measured by fast repetition rate fluorometry (FRRF). In the logarithmic growth phase, the *p*-value was high (around 0.3, see Fig. 4), indicating that PSII centres share a common antenna; this interconnection was lost in the stationary phase (*p*<0.1 in Fig. 4). At the same time, the effective antenna size of PSII significantly increased by 25% from *G. theta* cultures growing in logarithmic phase (σ_PSII_ of ~330 Å^2^) to stationary phase (σ_PSII_ of ~430 Å^2^) (Fig. 4); more chlorophyll molecules were effectively connected with PSII in the stationary phase.

**Fig. 5.**
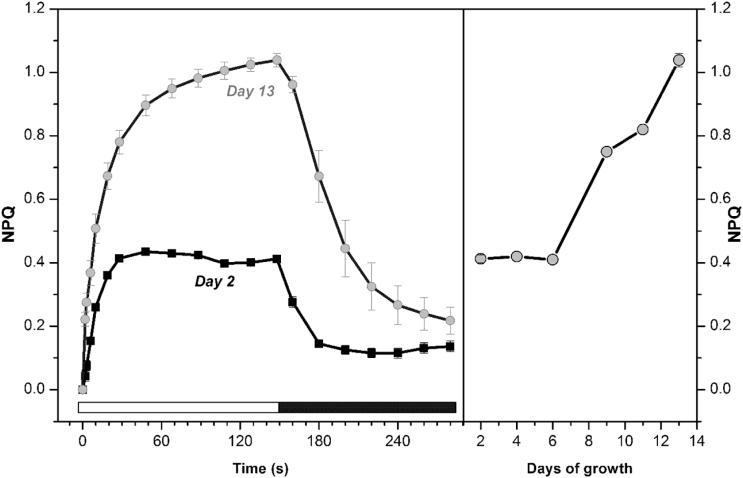
Non-photochemical quenching capacities of *G. theta* cells in dependence to the age of the culture. (A) The extent of NPQ of *G. theta* cells in a logarithmic phase of growth (Day 2, black line) and stationary phase of growth (Day 13, grey line) during 150 s of exposition to strong blue actinic light (750 µmol photons m^−2^ s^−1^; see white bar) and following 150 s of dark recovery (see black bar). (B) NPQ along the growth curve. NPQ was measured after 10min low light acclimation (2 µmol photons m^−2^ s^−1^) and calculated according to the Stern–Volmer formula as NPQ $=(F_{\text{m}}-F_{\text{m}}´)/F_{\text{m}}$. Data represent average and standard deviation of three biological replicates.

**Fig. 6.**
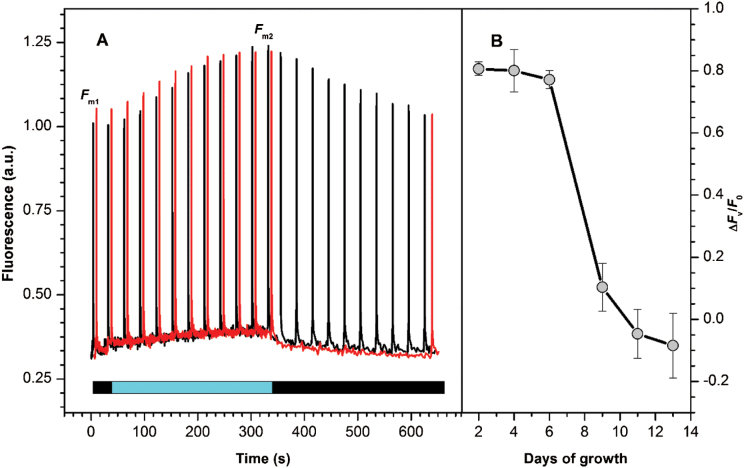
State-transition-like behavior of *G. theta* cells in dependence to the culture age. (A) Changes in maximal fluorescence during dark/light/dark transition. *G. theta* cells were dark adapted for 20min to measure the F_m1_ value, and then exposed to low blue light (see blue bar) to receive the F_m2_ value. Reversibility of this process was confirmed by a second dark period (black bar starting at 350 s). B. Difference in variable fluorescence F_v_ in samples exposed to low white light (2.5 µmol photons m^−2^ s^−1^) or dark adapted at different time points. F_v_ was normalized to the density of the culture with the help of the minimal fluorescence value F_0_; $\Delta F_{\text{v}}/F_{0}$ was calculated as $F_{\text{v}}(\text{LL})/F_{0}(\text{LL})-F_{\text{v}}(\text{D})/F_{0}(\text{D})$.

**Fig. 7.**
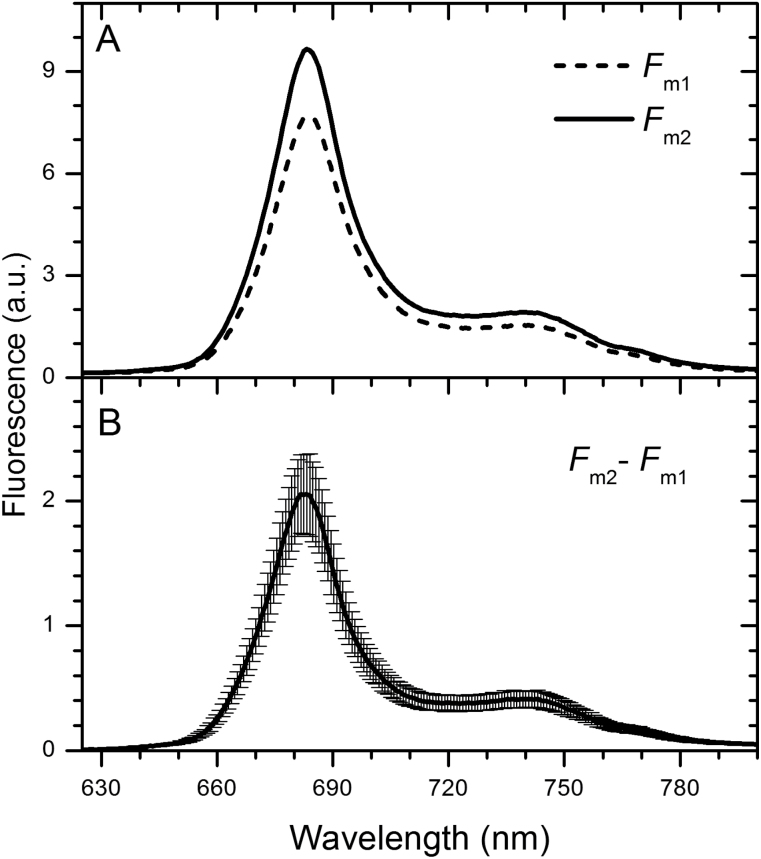
Changes in RT fluorescence emission spectra of cells exposed to low blue light. (A) Spectra of maximal fluorescence after 20min dark adaptation (F_m1_, dashed line) following exposure to low blue light for 5min (7 µmol photons m^−2^ s^−1^) (F_m2_; solid line). Representative curves are displayed. (B) Difference spectrum of F_m2_ and F_m1_. Presented curve is an average and SD from eight independent measurements.

Detailed analysis of NPQ in *G. theta* cultures was performed with respect to the culture age (Fig. 5B). Surprisingly, in the logarithmic phase, *G. theta* cells showed rather low NPQ (below 0.5, see Fig. 5A, Day 2), while in the stationary phase NPQ steadily increased (Fig. 5A, Day 13). These results are in contrast to NPQ measurements performed in *R. salina*, which displayed quite high NPQ values (~1.4 at Day 2, see Supplementary Fig. S1, available at *JXB* online) already in very young cultures. Further, the presence of state transition was quantified in *G. theta* at different culture ages. During logarithmic growth phase, characterized by low NPQ, a phenomenon similar to state-transitions was detected in *G. theta* cells (Fig. 6A); pretreatment with low-light (2 µmol photons m^−2^ s^−1^) was required to reach maximal fluorescence. When dark-adapted *G. theta* cells were exposed to low-intensity blue light (7 µmol photons m^−2^ s^−1^ for 300 s) the fluorescence increased, this process was reversed in darkness (Fig. 6A). This dark recovery was also observed during treatment with saturating blue flashes (see red curve in Fig. 6A), therefore it was independent of fluorescence quenching. Instead, the initial fluorescence increase, *F*_m_, in low blue light, was attributed to state 2 to state 1 transition; the following decrease in darkness then corresponded to state 1 to state 2 transition. State 2 to state 1 transitions were observed in *G. theta* only after exposure to low-intensity blue light, green light had no effect, neither on state 2 to state 1 transition nor on its recovery (see Supplementary Fig. 2A, 2B). Thus, state transitions in *G. theta* are exclusively controlled by chlorophyll-binding proteins and not by the lumen-located phycoerythrin antenna. Room temperature fluorescence emission spectra were recorded on dark-adapted cells either exposed to blue light for 5min or kept in darkness (Fig. 7). Dark-adapted cells had a substantially lower maximal variable fluorescence (*F*_m_) (see Fm_1_ in Fig. 7A). The difference spectra between F_m1_ in State 2 and F_m2_ in State 1 (Fig. 7B) displays a pronounced increase of chlorophyll *a* emission in F_m2_ originating from PSII. Interesting to note, state transitions were only observed in *G. theta* cultures in the logarithmic growth phase (Figs 6A, B and 7 and Supplementary Fig. S2); while these low light-induced changes in variable fluorescence disappeared in the stationary phase (after Day 6, see ΔF_v_/F_0_ in Fig. 6B), NPQ increased (Fig. 5B).

### Presence of state transitions correlates with maximal photosynthetic performance in *G. theta*

**Fig. 8.**
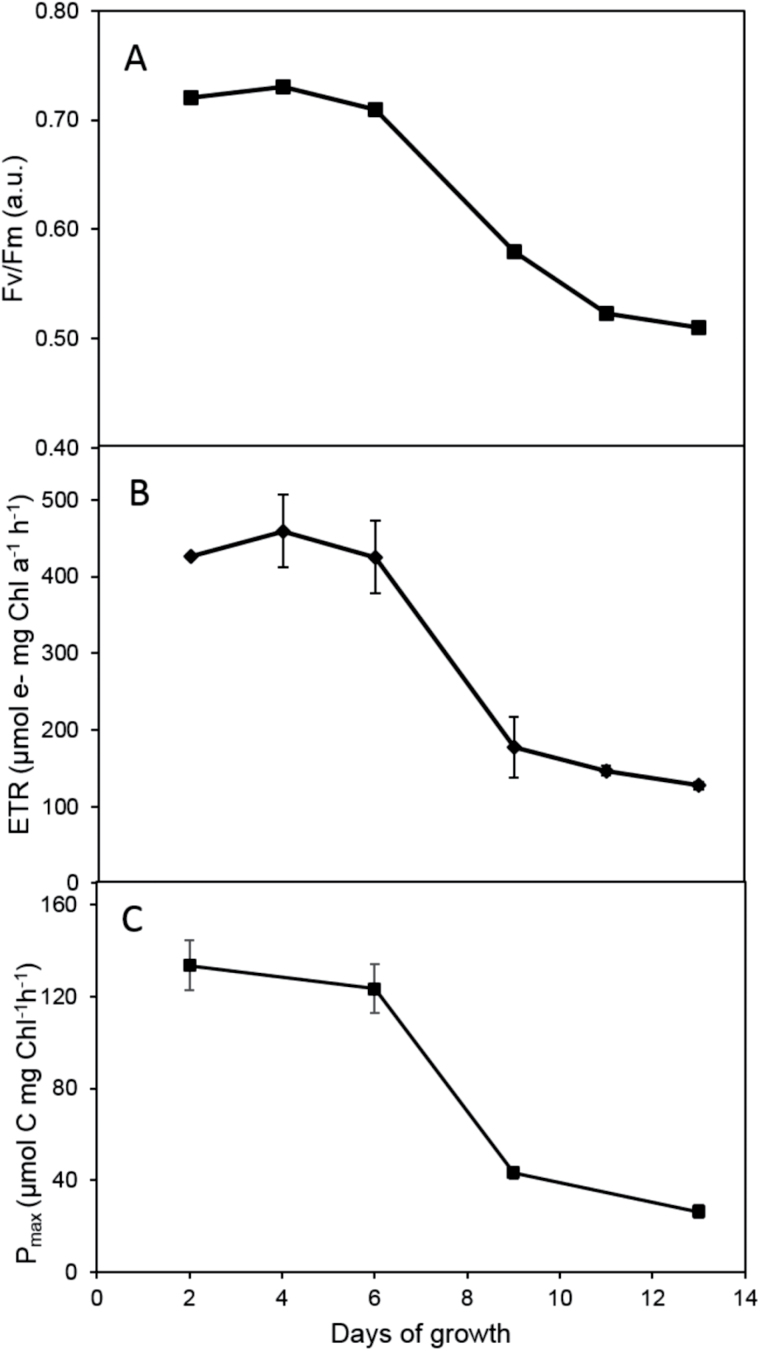
Photosynthetic parameters of *G. theta* cultures grown under standard conditions. (A) The maximal efficiency of PSII photochemistry (F_v_/F_m_). (B) Electron transport rate (ETR) within Photosystem II. C. CO_2_ fixation rate measured as P_max_.

Growth phase-dependent photosynthetic activity of primary (PSII efficiency, electron transport rate) and secondary (CO_2_ fixation) photosynthetic reactions was analysed in *G. theta* cultures. During lag and logarithmic growth phases, maximal efficiency of PSII photochemistry (F_v_/F_m_) was detected (Fig. 8). It then gradually declined as the culture progressed to the stationary phase (Day 6–9, Fig. 1). F_v_/F_m_ decreased by 30% from its value of 0.7 during logarithmic phase (up to Day 6) to 0.5 in stationary phase (day 13) (Fig. 8A). The absolute electron transport rate (ETR), quantifying the maximal rate of electron generation in PSII and thus the maximal capacity of primary photosynthetic reactions rapidly declined between Days 6 and 9, when the cultures were entering the stationary phase (Fig. 8B). Within the stationary phase (Days 9–13) an additional small gradual decline of ETR was observed. Changes in ETR correlated with the overall capacity of photosynthetic carbon fixation measured from ^14^C incorporation rate (Fig. 8C). As expected, carbon fixation rapidly declined between Days 6 and 9, consistent with the lowered PSII maximal efficiency (Fig. 8A) and electron transport rate (Fig. 8B). The transition from logarithmic to stationary phase was therefore accompanied by pronounced changes in the overall photosynthetic capacity.

## Discussion

In the present study, it was shown that the cryptophyte alga *G. theta* is able to perform state transitions and non-photochemical quenching (NPQ) to efficiently modulate photosynthesis. Interestingly, *G. theta* uses these two mechanisms in different growth stages. In the logarithmic growth phase, state transitions are performed, photosynthetic activity as well as CO_2_ fixation are high, cells contain a high amount of chlorophyll, meanwhile NPQ is very low. However, when the cells enter the stationary phase, instead of state transition they use NPQ; the cells grow in size, meanwhile their chlorophyll content per cell drops. The reason for this separation between the two types of photo-protection, which has not been reported in higher plants, is not known at present time. This work demonstrates that *G. theta* represents a chromalveolate algae that is able to perform state transitions.

### State transitions

Previously it has been shown that the cryptophyte alga *R. salina* can perform fast and flexible NPQ (qE type) in the logarithmic growth phase (Kaňa *et al.*, 2012b). Also *G. theta* is capable of performing NPQ (Funk *et al.*, 2011), however, this mechanism is restricted to the stationary growth phase. In the logarithmic growth phase, under conditions when *R. salina* uses NPQ, *G. theta* performs instead state transitions to regulate the amount of energy arriving at the reaction centers. State transitions dependent on the culture age have been reported before for the chromophyte alga *Ochromonas danica* (Gibbs and Biggins, 1989). Dark adapted *G. theta* cells were in the low-fluorescence state 2, transition to the high-fluorescence state 1 was induced by low intensity blue (7 µmol m−^2^ s^−1^) or very low intensity white light (2 µmol m^−2^ s^−1^; not shown). At first view, the mechanism of state transitions performed by *G. theta* resembles the process observed in cyanobacteria (Campbell *et al.*, 1998); when dark-adapted cyanobacterial cells are irradiated with low blue light (the light preferentially absorbed by PSI) PQ oxidation increases due to their high PSI:PSII ratio. Antenna redistribution towards PSII results then in the high-fluorescent state 1 (for review see e.g.Kirilovsky *et al.*, 2014). This process is observed in dark-adapted cyanobacteria cells, when respiration leads to a reduced PQ pool (Mullineaux and Allen, 1990), causing efficient energetic coupling of the phycobilisomes to PSI (see e.g. Papageorgiou *et al.*, 2007; Kaňa *et al.*, 2012a). Organization of the light-harvesting antenna in cryptophytes, however, is very different from the one in cyanobacteria. The large cyanobacterial phycobilisomes are stromal protein complexes and transfer light energy directly to the reaction centres; state transition seem to be connected with phycobilisome mobility (Joshua and Mullineaux, 2004; Kaňa *et al.*, 2014) or with their slight re-arrangement state 2 is stimulated by phycobilisome-absorbed orange light (see Kirilovsky *et al.*, 2014)) for recent review). Cryptophytes, on the other hand, contain lumen-located phycoerythrin (PE) biliproteins as well as intrinsic chlorophyll *a/c* antenna complexes. The PE antenna seems to be rigid and immobile (Kaňa *et al.*, 2009). In *G. theta* green light, absorbed by phycoerythrin, did not induce state transition like changes (see Supplementary Fig. S2); state transitions were solely induced by light absorbed by chlorophylls (blue light, Fig. 6A) of the membrane integrated Chl *a/c* antenna. Since the low-temperature (77K) emission peaks of PSI (F705) and PSII (F694) in cryptophytes are very close to each other (Supplementary Fig. S3), no antenna redistribution was revealed between PSI and PSII during state transitions. Still, the room temperature measurements of maximal fluorescence (Fig. 7) clearly demonstrate that thylakoid membranes reorganize during state transitions and involve the intrinsic Chl *a/c* binding antennae, although the extent to which the Chl *a/c* antennae of *G. theta* are preferentially coupled to PSII (state 1, exposure to blue light) or to PSI (state 2, darkness) cannot be estimated. In higher plants and green algae, state transitions involve phosphorylation of the light harvesting antennae (Minagawa, 2011). This is in contrast to red algae, where the light induced protein phosphorylation patterns were the same in state I and state II (Biggins and Bruce, 1989) and inhibitors of phosphorylation had no effect on state transitions (Delphin *et al.*, 1995). In cryptophytes phosphorylation of the Chl *a/c* binding intrinsic antenna has been demonstrated in *Chroomonas sp*. (Janssen and Rhiel, 2008). The role of phosphorylation for state transitions of cryptophytes will have to be demonstrated in future experiments.

The PE antenna of cryptophytes was recently shown to be extremely efficient in light harvesting due to their quantum coherence (Collini *et al.*, 2010). Even though state transitions in *G. theta* cultures growing in the logarithmic phase were light absorbed by the intrinsic chlorophyll *a/c* antenna, they induced re-arrangements of the PE antennae within the thylakoid lumen (Fig. 3): in dark-adapted logarithmically growing cells bilins with a ‘typical’ emission maximum at 589nm (F589) were observed as well as an additional pool displaying the unusual maximum at 576nm (F576). Upon low-light treatment (Fig. 3B), however, F576 decreased. In the stationary culture phase, when state transitions were absent, F576 was negligible and only F589 was observed, independent of the pretreatment (see Day 9 in Fig. 3A). Using *in vitro* measurements on isolated PE, a blue shifted emission maxima (like F576) was attributed to a loss of exciton splitting in exciton-coupled pairs of bilins (MacColl *et al.*, 1999b). These coupled bilin-pairs were described to be the lowest-energy chromophores receiving energy from other bilins and more importantly, delivering the energy to the next chromo-protein (for more details see MacColl *et al.*, 1999a). The exciton-coupling/splitting of the bilin pairs therefore might control the energy transfer from the PE antenna to the chlorophyll *a/c* antenna and explain the observed changes in the F589/F576 ratio. Blue light seems to induce excitonic coupling of PE to the intrinsic chlorophyll *a/c* antennae, which in turn is coupled to PSII as evident from the increased maximal fluorescence in state 1 (Figs 6 and 7). Such a mechanism would not require any physical movements of the PE biliproteins, very small re-arrangements of the antenna proteins would be sufficient. In the cryptphytes *R. salina* (Kaňa *et al.*, 2009) and *Cryptomonas ovata* (Snyder and Biggins, 1987) the luminal-located PE antennae were observed to be immobile.

### Non-photochemical quenching replaces state transitions in stationary growth phase

While state transitions disappeared in the stationary growth phase of a *G. theta* culture (Fig. 6B), photoprotective NPQ increased (Fig. 5B). At the same time the PE content per cell diminished in the stationary phase (Fig. 2B), most likely the degraded phycobiliproteins were used as an additional N source (Lewitus *et al.*, 1990). Owing to the decreased amount of PE in the stationary phase, NPQ in *G. theta* can be assigned to the Chl *a/c* antenna, similar to *R. salina* (Kaňa *et al.*, 2012b). However, in *R. salina* NPQ was found to be high already in very young cultures (Supplementary Fig. S1), while NPQ in *G. theta* was restricted to the stationary growth phase (Fig. 5B). ^14^C incorporation experiments measured at increasing light intensities have shown that NPQ in *R. salina* occurs when the Calvin–Benson cycle is saturated (Kaňa *et al.*, 2012b). In *G. theta* the CO_2_ fixation rate was drastically decreased in the stationary phase (Fig. 8C). Under these conditions, ATP regeneration is expected to slow down and as a consequence the lumen should become acidified. In *R. salina* it was shown that lumen acidification triggers NPQ, low pH induced the quenching state in the Chl *a/c* antenna via their reversible protonation (Kaňa *et al.*, 2012b). Here, it is proposed that also in *G. theta* NPQ is a pH-dependent process resembling the described mechanism in *R. salina*.

The photo-protective capacity seems to be higher in *R. salina* (Supplementary Fig. S1) compared with *G. theta* (Fig. 5). Differences in the photo-protective capacity in diatoms have been explained by their adaption to different ecological niches (Lavaud *et al.*, 2007; Lavaud and Lepetit, 2013). Analysing the phylogenetic relation of the two cryptophyte species based on SSU rDNA sequences (Hoef-Emden, 2008), indeed they were grouped into two distant evolutionary clades. Moreover they occupy different ecological niches with *R. salina* being estuarine (Hammer *et al.*, 2002), while *G. theta* was isolated from coastal regions (Rappé *et al.*, 1998). Estuarine species are known to possess a higher and more flexible capacity for photoprotection than oceanic and coastal ones (Strzepek and Harrison, 2004; Lavaud *et al.*, 2007). The two algae therefore perform different strategies: to survive in fluctuating and dynamic light conditions *R. salina* requires efficient NPQ, while *G. theta* rather optimizes its light-harvesting capacity for efficient photosynthesis using state transitions; NPQ is only induced as a feedback reaction, when photosynthesis is down-regulated in the nutrient-limited stationary phase.

### Physiological role of state transitions in *G. theta*

Equilibrating the distribution of excitation energy between the photosystems, state transitions are known to be important not only under limiting light conditions, but also to protect against photoinhibition by minimizing PSII antenna size at high light intensities (Minagawa, 2011). While they are less significant in higher plants (Lunde *et al.*, 2003), as much as 80% of the LHCII was found to be mobile during state transitions of green algae (Finazzi *et al.*, 2001; Drop *et al.*, 2014). In cyanobacteria, their importance in photoprotection still is a matter of debate (for recent review see e.g. Kirilovsky *et al.*, 2014), however, they maximize the light-harvesting efficiency during low light exposure (Mullineaux and Emlyn-Jones, 2005). In green algae it was observed that part of the mobile PSII antenna is detached from PSII in state 2 and without docking to PSI is switched into a quenched state (Ünlü *et al.*, 2014). Such coupling/decoupling of the (phycobilins) antenna has also been proposed for cyanobacteria (Kirilovsky *et al.*, 2014). Here it is proposed that even in *G. theta* the antennae seem to attach to the photosystems under low light conditions and detach under excess light. While these state transitions are preferentially triggered in the Chl *a/c* antenna, they involve both, the membrane integrated Chl *a/c* antennae as well as the PE antennae in the thylakoid lumen. The process optimizes photosynthesis in the logarithmic growth phase and thus maximizes photosynthetic capacity (see PSII, ETR and Pmax in Fig. 8) under light-limiting conditions. It therefore represents a physiological advantage of *G. theta in situ*.

## Supplementary data

Supplementary data are available at *JXB* online.

Fig. S1. Non-photochemical quenching capacities of *R. salina* cells during logarithmic phase of growth.

Fig. S2. Effect of green light on state-transitions of *G. theta* cells.

Fig. S3. Low temperature (77 K) fluorescence emission of *G. theta* cells after excitation of chlorophylls at 436nm.

## Funding

This work was supported by the Swedish Energy Agency (2012–005889) (to C.F.), Umeå University and the Artificial Leaf and Solar Fuel Project (KAW 2011-0055) (to O.C., WP.S. and C.F.). E.K, O.P. and R.K. were supported by projects GAČR P501/12/G055 and GAČR P501/12/0304 financed by the Czech Science Foundation and by the project Algatech (CZ.1.05/2.1.00/03.0110) and Algatech Plus (MSMT LO1416) provided by Czech Ministry of Education, Youth and Sport.

## Supplementary Material

Supplementary Data
