# Supplementary material for: Presence of state transitions in the cryptophyte alga Guillardia theta
Source: J Exp Bot. 2015 Aug 6;66(20):6461–70. doi: 10.1093/jxb/erv362 (PMC4588893; doi:10.1093/jxb/erv362)
Supplement: Supplementary Data [file supp_66_20_6461__index.html]

Presence of state transitions in the cryptophyte alga Guillardia theta — Presence of state transitions in the cryptophyte alga Guillardia theta — Supplementary Data 

# Presence of state transitions in the cryptophyte alga *Guillardia theta*

## Supplementary Data

Data files

- Supplementary Data - Supplementary Data
