# Supplementary material for: Presence of state transitions in the cryptophyte alga Guillardia theta
Source: J Exp Bot. 2015 Aug 6;66(20):6461–70. doi: 10.1093/jxb/erv362 (PMC4588893; doi:10.1093/jxb/erv362)
Supplement: Supplementary Data [file supp_erv362_jexbot150201_file001.pdf]

Journal of Experimental Botany, “Presence of state transitions in the cryptophyte alga *Guillardia theta”*

Otilia Cheregi, Eva Kotabová, Ondřej Prášil, Wolfgang P. Schröder, Radek Kaňa, Christiane Funk.

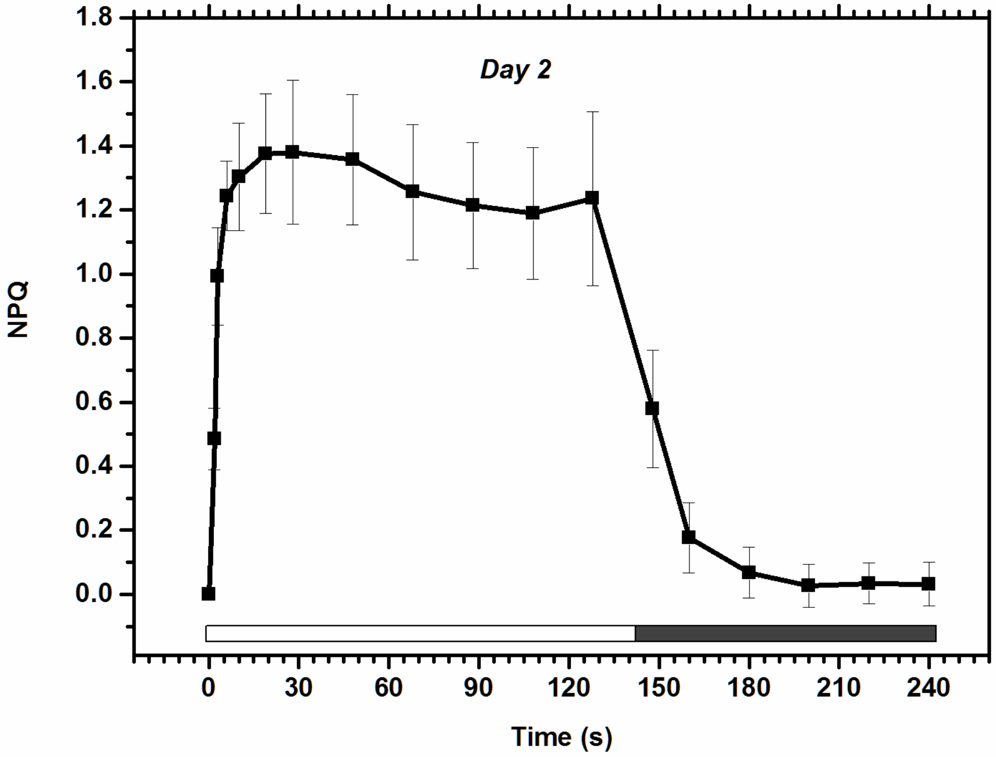

Supplementary Figure S1. Non-photochemical quenching capacities of *R. salina* cells during logarithmic phase of growth. The extent of NPQ in a logarithmic growth phase (day 2, black line) during exposure to strong blue actinic light (750 µmol photons m^{-2} s^{-1}; white bar) for 140 s followed by dark recovery for 100 sec (black bar). NPQ was measured 10 min after dark adaptation and calculated as decribed in Material and Methods. Data represent an average and standard deviation for n=3.

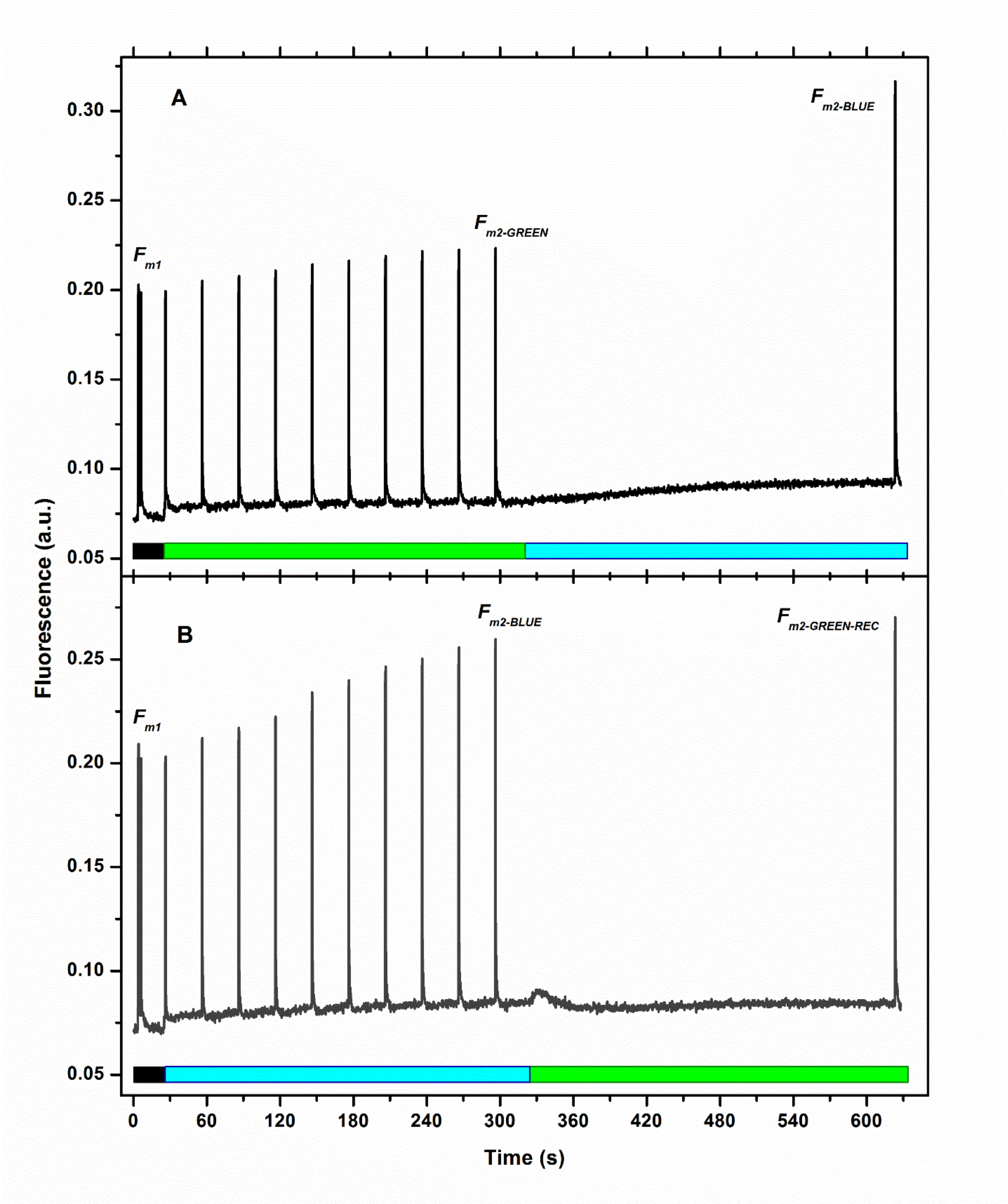

Supplementary Figure S2. Effect of green light on state-transitions of *G. theta* cells **A.** State 2 to state 1 transition. *G. theta* cells were dark adapted for 20 minutes to receive the F_{m1} value, and then exposed to low green light (8 µmol m^{-2} s^{-1}, 520 nm, green bar) to measure F_{m2}-GREEN. The

maximal value of F_{m2}-BLUE induced by blue light was then measured after 330 s (blue bar). **B.** State 1 to state 2 transition. *G. theta* cells were dark adapted for 20 minutes to receive the F_{m1} value, then exposed to low blue light (7 µmol m^{-2} s^{-1}, 464 nm, blue bar) to measure the F_{m2}-BLUE value. The effect of green light on the recovery from state 1 to state 2 was measured after 330 s (green bar) to receive F_{m2}-GREEN-REC.

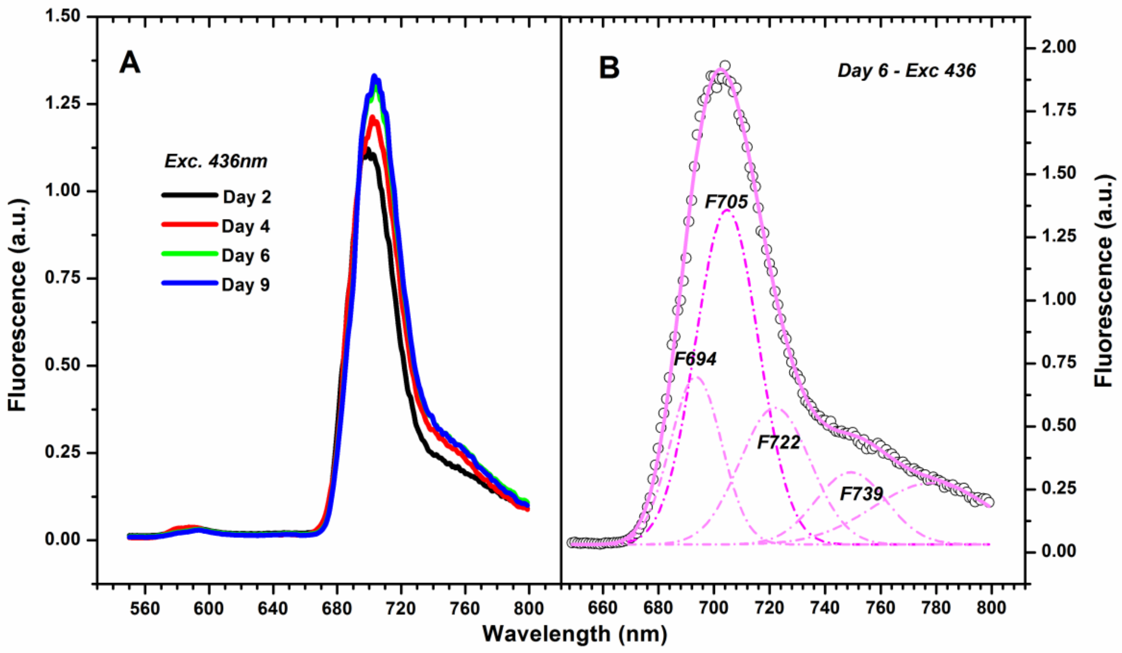

Supplementary Figure S3. Low temperature (77 K) fluorescence emission of *G. theta* cells after excitation of chlorophylls at 436 nm. **A.** Spectra of the culture in logarithmic and in stationary growth phase. **B.** Deconvoluted spectrum obtained of cells in logarithmic growth phase (day 2) displaying the four main fluorescence emission maxima (F694, F705, F722 and F739).
